# Reducing stillbirths in Ethiopia: Results of an intervention programme

**DOI:** 10.1371/journal.pone.0197708

**Published:** 2018-05-30

**Authors:** Bernt Lindtjørn, Demissew Mitike, Zillo Zidda, Yaliso Yaya

**Affiliations:** 1 Centre for International Health, University of Bergen, Bergen, Norway; 2 Gidole Hospital, Gidole, Ethiopia; 3 Arba Minch Hospital, Arba Minch, Ethiopia; John Hunter Hospital, AUSTRALIA

## Abstract

Previous studies from South Ethiopia have shown that interventions that focus on intrapartum care substantially reduce maternal mortality and there is a need to operationalize health packages that could reduce stillbirths. The aim of this paper is to evaluate if a programme that aimed to improve maternal health, and mainly focusing on strengthening intrapartum care, also would reduce the number of stillbirths, and to estimate if there are other indicators that explains high stillbirth rates. Our study used a “continuum of care” approach and focussed on providing essential antenatal and obstetric services in communities through health extension workers, at antenatal and health facility services. In this follow up study, which includes the same 38.312 births registered by community health workers, shows that interventions focusing on improved intrapartum care can also reduce stillbirths (by 46%; from 14.5 to 7.8 per 1000 births). Other risk factors for stillbirths are mainly related to complications during delivery and illnesses during pregnancy. We show that focusing on Comprehensive Emergency Obstetric Care and antenatal services reduces stillbirths. However, the study also underlines that illnesses during pregnancy and complications during delivery still represent the main risk factors for stillbirths. This indicates that obstetric care need still to be strengthened, should include the continuum of care from home to the health facility, make care accessible to all, and reduce delays.

## Introduction

Between 1.7 and 2.5 million (median estimate 2.1 million) stillbirths occur every year, and most of them in developing countries [1]. Ethiopia was in 2009 among the ten countries in Asia and Sub-Saharan Africa that accounted for 67% of all stillbirths [2]. Although many countries have managed to reduce stillbirths, Sub-Saharan African countries still have high stillbirth rates [1, 3], and even if the Millennium Development Goals focused on maternal and child health, the decline in stillbirths and neonatal deaths rates were slow [4, 5].

Although the causes of stillbirths are not known in detail in Ethiopia, verbal autopsies from rural communities in Africa show that stillbirths are associated with infection, prolonged labour, antepartum haemorrhage, preterm delivery, cord complications and accidents [6]. However, the true causes of stillbirths remain uncertain in most low-income countries [7]. Most of the earlier research on stillbirth rates in Ethiopia are based on institutional based studies and demographic surveillance sites [5, 8]. A recent study from Ethiopia showed the stillbirth rate was lower among women receiving prenatal care, but delivery in a health facility did not reduce the risk of stillbirth compared with stillbirth rates among women delivering at home [9]. Other intervention studies failed to show that women served by health centres with a maternal health package had fewer stillbirths compared with health institutions without such an intervention [10].

Based on information about poor maternal and new-born services in Ethiopia [11], we started in 2008 an implementation study in south-west Ethiopia. This was a part of many years of collaboration between health institutions in south-west Ethiopia and Norwegian organisations. The aim of the project was to strengthen the health institutions and improve the population coverage of essential maternal and neonatal health services. The main project aim was to reduce maternal deaths, but also stillbirths and neonatal deaths [12]. With the Ministry of Health of Ethiopia, we designed a project to strengthen the health care system [13]. Particular emphasis was given to upgrading existing institutions so they could improve antenatal care, carry out Basic Emergency Obstetric care (BEmOC) and Comprehensive Emergency Obstetric care (CEmOC).

Using a population-based birth registry, we collected information about maternal and neonatal deaths, and stillbirths (see methods for more details) [14]. This intervention programme resulted in a 54% decline in maternal deaths over four years [13]. This current paper is an extension of the article on reducing maternal deaths [13]. In line with the implementation project’s goals, and using the same data used from our published study on maternal deaths, all the background information in our previous publication are relevant for this paper [13]. The aim of this paper is to evaluate if a programme that mainly focused on strengthening intrapartum care also would reduce the number of stillbirths, and to assess if there are other indicators that explain high stillbirth rates. Thus, this study is relevant for efforts to operationalise health packages that could reduce stillbirths.

## Methods

### Ethics statement

This research was an implementation study, and the work done was a part of the routine work of the hospitals, health institutions and health extension workers in the area. Birth and birth-outcome registration is part of the routine work of the HEWs in Ethiopia, which is acknowledged by the government. For the research part, the Institution Review Board for Health Research of Southern Nations Nationalities and Peoples’ Regional State in Ethiopia (SJ42/6677), and the Regional Committee for Health Research Ethics of North Norway (2011/2495/Rek nord), approved the study. Personal identifiers were removed from the stored data used for research.

### Study area and population

This study on decline of stillbirths is a follow up of an implementation study that aimed at improving maternal deaths [13]. This intervention study was done in the three same districts (woredas) in South-west Ethiopia in the Southern Nations, Nationalities and Peoples' Region. The data collection on information such as recruitment, data collection, and data analysis are presented in an earlier study [13].

As written in our earlier papers, “this study involved people from different cultural backgrounds and three ethnic groups in south Ethiopia (Gamo, Zeisse, and Derashe). The study population comprises 38.312 deliveries in the Dirashe, Arba Minch Zuria and Bonke woredas (sub provinces). In 2010, about 170.000 people lived in Bonke. This woreda had no hospital providing CEmOC during the study; so, people in need of such services had to travel to the nearest hospital, the Arba Minch Hospital, found about 100 kilometres from Bonke.

At the time of our study, roughly 380.000 people lived in the Arba Minch Zuria Woreda, and the district had one large hospital. In 2010, about 142.000 people lived in Dirashe. This district is served by Gidole Hospital, which has a well-functioning maternity waiting area where mothers with high-risk pregnancies are referred to- and observed until delivery [15]. Our study involved people from different cultural backgrounds and ethnic groups.

The area we conducted the studies in were similar in demographics, health services, road access, and economic structure with most rural communities in Ethiopia. To register births and stillbirths, we developed a birth registry using the existing community health workers, in which household visit is their routine activity, with priority given to households with pregnancies and births. The health extension workers who performed the birth registration and identified maternal deaths are distributed in the same proportion to the population and have the same training background, all are females and all have similar working conditions in the country” [13, 16].

### Implementation programme

Our implementation project worked closely with the health extension programme which represents a responsive health delivery system to people in rural areas and aims to ensure equitable access to disease prevention and control, family health service (including maternal and child health), hygiene and environmental sanitation, and health education [17]. The health extension workers (HEWs) are women, receive a one-year training, and are responsible for the health services for 500–1000 households. The HEWs who performed the birth registration and identified maternal and neonatal deaths, and stillbirths are distributed in proportion to the population size in the communities and have the same training background; all are females and all have similar working conditions in the country. Thus the HEWs can conduct a birth registration of high coverage and optimum quality, and can classify pregnancy and birth results in any rural community in Ethiopia [16].

With the Ministry of Health, we designed a project to strengthen the health care system [12, 13]. A particular emphasis was given to upgrading existing institutions so they could carry out antenatal care (ANC), BEmOC and CEmOC. These health institutions were upgraded by training non-clinical physicians and midwives by providing the institutions with essential and basic equipment, and by regular monitoring and supervision by staff competent in emergency obstetric work. During the intervention period, the number of institution carrying our comprehensive obstetric emergency services, and neonatal intensive care units increased substantially. Details about the number and types of institutions before and after the interventions has previously been published [13].

WHO defines antenatal care (ANC) as “the care provided by skilled health-care professionals to pregnant women and adolescent girls in order to ensure the best health conditions for both mother and baby during pregnancy. The components of ANC include: risk identification; prevention and management of pregnancy-related or concurrent diseases; and health education and health promotion [18]”. In our study area, most of ANC was carried out by HEWs. Even if they are not defined as skilled health workers, they have a one-year training which includes basic ANC elements.

The details of this work, including number of institutions and quality assessment of the work of the non-clinical physicians doing caesarean sections has previously been outlined [13]. The scope of the work, and the basic and essential equipment was defined as used for BEmOC and CEmOC services and outlined in WHO manuals [19, 20].

As outlined in details in our previous paper on reducing maternal deaths [13], “the mean number of antenatal visits for each woman was 2.6 (interquartile range (IQR) 2–4). However, this varied between the districts with an average of 3.0 (IQR 2–4) visits in Dirashe, 2.2 (IQR 1–3) visits in Bonke and 2.7 (IQR 2–4) visits in Arba Minch Zuria. Between 2010 and 2013, the percentage of pregnant women who attended four or more pregnancy controls improved by 28% in Dirashe by 16% in Arba Minch and by 17% in Bonke. Similarly, the number of women referred to an institution with a skilled birth attendant increased most in Dirashe, followed by Arba Minch, and least in Bonke.”

The aim of the implementation project was to assure that each health facility had obstetric services available 24 hours a day and seven days a week and were staffed by skilled health professionals. “An Emergency Obstetric Care (EmOC) facility refers to whether an institution is fully functioning as a BEmOC or CEmOC facility [21]. We defined the functioning by nine signal functions: administering parenteral antibiotics, administering parenteral oxytocic drugs, administering parenteral sedatives, manual removal of the placenta, removal of retained products of conception, assisted vaginal delivery (vacuum or forceps delivery). Institutions, which in addition to these signal functions could do caesarean sections and have a blood transfusion service, were defined as CEmOC facilities. All institutions received basic equipment, and training in neonatal resuscitation. Each of the institutions were supervised, at least once every quarter, to monitor the progress of the work” [13].

### Study design outcome measures

In this implementation study, the primary outcome measure was stillbirths and we used the definition recommended by WHO as “a baby born with no signs of life at or after 28 weeks' gestation” and measured as number of stillbirths per 1000 births [22]. The assessment of gestational age was based on a history of the last menstruation period. We also measured the use of antenatal controls, and other explanatory variables included distance to institution, literacy of both the husband and of delivering mothers, history of previous pregnancies and deliveries, and whether any illness had occurred during the pregnancy or if the delivery was complicated. Illness during pregnancy and delivery was based on if the HEWs defined the pregnant or delivering mother to have had an illness. A complicated delivery was defined as a delivery that lasted too long, if the HEW or skilled birth attendant assessed the bleeding to be too heavy, or the mother was referred to an institution where the mode of delivery was not a normal cephalic delivery.

Because the interventions we used are believed to be effective [23], we considered it unethical to introduce a control area without access to such interventions. Our study thus analyses trends in the use of interventions and simultaneously occurring reductions in stillbirths.

### Birth registry

Before starting the health interventions, we developed a community based birth registry. The aim of the registry was to monitor whether maternal mortality declined, if the mothers were referred or not, where the babies were delivered and who helped the mother during the delivery. Using the skills of the Health extension workers (HEWs), we set up, field-tested, validated, and implemented a community-based birth registry to record births and outcomes such as maternal deaths, neonatal deaths and stillbirths [13, 14]. This population-based birth registration system was validated comparing the community birth registration with cross-sectional surveys, the sisterhood method and with the institutional-based registration of maternal deaths [14, 16]. Our conclusion was that the birth registration done by health extension workers provides a valid, community-based measurement of maternal deaths [16]. However, even if the maternal mortality measurements were found to be valid using the birth registry, we found that the population coverage the birth registrations were about 72%, with women living furthest away having lower coverage in the birth registry [14, 16].

Although we did not validate to register stillbirths as we did for maternal and neonatal deaths, we retrospectively recorded 226 stillbirths over a five-year period (2007–2011) [14]. On the average, that is about 45 stillbirth deaths per year, and in agreement with the first assessment in 2010 in Bonke [24].

Each month, nursing supervisors visited the health post, checking the registry for completeness, supervising the health extension worker and taking a copy of the registry book to the project office. Our birth registration contains about 3½ years (from 2010 to mid-2013) of quality assured, and uninterrupted data collection and valid data. In late 2013, the birth registries were transferred to the local government system. This caused an interruption that resulted in data that could not be fully quality assured, and we therefore only included data in which supervisory nurses checked the quality of the birth registration.

### Data management and analysis

Data were entered into a computer using SPSS software (SPSS Inc. Chicago, IL), and the data were later checked for completeness and errors, and the paper forms could be returned to the health post for further checks. For the analysis, we calculated yearly stillbirth incidence rates, and odds ratios (problem during delivery, illness during pregnancy, sex of baby, age of the mother, father education, availability of CEmOC services, use of 4 or more antenatal visits) and logistic regression for multivariable analysis. The results are presented with P values and crude and adjusted Odds ratios with 95% confidence intervals.

## Results

The rate of stillbirth decreased (Table 1) during the implementation period from 2010–2013 by 46%, from 14.5 till 7.8 per 1000 births (X^2^ for trend = 24.4, P<0.001. The decrease occurred in areas with good access to CEmOC (Dirashe and Arba Minch Zuria woredas), but not in a district where only BEmOC services was available (Bonke) (Table 1).

**Table 1:** Number of stillbirths (per 1000 births with 95% confidence intervals) for each district and year.

District	Year	Number stillbirths	Total births	Stillbirth rate with 95% CI
Dirashe	2010	37	3132	11.8 (8.4–16.1) *
	2011	16	2688	6.0 (3.5–9.4)
	2012	6	2396	2.5 (1.0–5.4)
	2013	6	2065	2.9 (1.2–6.3)
	Total	65	10281	6.3 (4.9–8.0)
Bonke	2010	47	3737	12.6 (9.3–16.6)
	2011	51	4747	10.7 (8.1–14.0)
	2012	49	4472	11.0 (8.0–14.3)
	2013	24	1224	19.6 (12.0–29.0)
	Total	171	14180	12.1 (10.4–14.0)
Arba Minch	2010	77	4252	18.1 (14.4–22 5) *
	2011	49	4523	10.8 (8.1–14.2)
	2012	24	3344	7.2 (4.8–10.5)
	2013	9	1732	5.2 (2.5–9.5)
	Total	159	13851	11.5 (9.8–13.4)
Total	2010	161	11121	14.5 (12.4–16.8) *
	2011	116	11958	9.7 (8.1–11.6)
	2012	79	10212	7.7 (6.2–9.6)
	2013	39	5021	7.8 (5.6–10.5)
	Total	395	38312	10.3 (9.4–11.4)

Test for linear or trend

* P<0.001

Of the 395 stillbirths recorded, 7.6 per 1000 births (247 of 32540 births) occurred during home deliveries, 5.6 (14 of 2489 births) at health posts, 20.4 (37 of 1813 births) at health centres and 65.4 (97 of 1384 births) at hospitals (Table 2). At hospitals, the stillbirth rates were lowest at hospitals in Dirashe and Arba Minch provinces, and it was higher for patients referred from Bonke to the Arba Minch hospital over 100 km away (35.0 per 1000 stillbirths in Dirashe, 67.0 in Arba Minch and 349.4 per 1000 births in Bonke). We did not find any association between increased risk for stillbirths and available road types (asphalt, gravel or no road for transportation). Table 2 also shows the stillbirth rates were highest among the youngest and oldest mothers, and among the primigravida and mothers with six or more deliveries.

**Table 2:** Stillbirth rates for place of delivery, literacy rate, and age of mother and father, and number of pregnancies.

Variable			stillbirth		Total	Stillbirth rate
			Yes	No		
Place of delivery (where?)						
	Dirashe	Home	32	8184	8216	3.9
		Health post	2	693	695	2.9
		Health centre	3	567	570	5.3
		Hospital	28	772	800	35.0
	Bonke	Home	117	12284	12401	9.4
		Health post	8	1308	1316	6.1
		Health centre	17	363	380	44.7
		Hospital	29	54	83	349.4
			171	14009	14180	
	Arba Minch	Home	98	11825	11923	8.2
		Health post	4	464	468	8.5
		Health centre	17	846	863	19.7
		Hospital	40	557	597	67.0
	All places	Home	247	32293	32540	7.6
		Health post	14	2465	2479	5.6
		Health centre	37	1776	1813	20.4
		Hospital	97	1383	1480	65.5
	Total		395	37917	38312	10.3
Mother literacy		Illiterate	294	27961	28255	10.4
		Grade 1–6	67	7572	7639	8.8
		Grade 7 and more	34	2384	2418	14.1
Father education		Illiterate	219	19820	20039	10.9
		Grade 1–6	114	12438	12552	9.1
		Grade 7 and more	62	5659	5721	10.8
Age of mother		Less than 20 years	35	2411	2446	14.3
		21–30 yrs.	254	26703	26957	9.4
		31–40 yrs.	104	8700	8804	11.8
		Over 40	2	103	105	19.0
Gravidae		First	85	6442	6527	13.0
		Gravidae 2–5	197	24290	24487	8.0
		Gravidae 6 or more	113	7182	7295	15.5

The number of delivering mothers who were referred to health centres and hospitals increased from 6.4% (709 of 11113 births) to 16.9% (838 of 4959 births) (Mantel-Haenszel chi-square for linear trend 408.34; P<0.001). Table 3 shows there was a decreasing trend of stillbirths among those who were referred to health institutions (heath centres and hospitals); from 91.7 to 25.1 per 100 births (Mantel-Haenszel chi-square for linear trend 40.16; P<0.001).

**Table 3:** Referral pattern for the three districts by year.

Year	Referred?	Stillbirth?		Total	Stillbirths
		Yes	No		per 1000 births
2010	Not referred	96	10308	10404	9.2
	Referred to HC	28	278	306	91.5
	Referred to Hospital	37	366	403	91.8
2011	Not referred	69	11191	11260	6.1
	Referred to HC	20	292	312	64.1
	Referred to Hospital	27	358	385	70.1
2012	Not referred	54	9304	9358	5.8
	Referred to HC	14	502	516	27.1
	Referred to Hospital	11	324	335	32.8
2013	Not referred	18	4103	4121	4.4
	Referred to HC	5	492	497	10.1
	Referred to Hospital	16	325	341	46.9
Total	Not referred	237	34906	35143	6.7
	Referred to HC	67	1564	1631	41.1
	Referred to Hospital	91	1373	1464	62.2
Total		395	37843	38238	10.3

The combined effect of seven variables (complication during delivery as assessed by health extension workers, illness during pregnancy or abnormal childbirth, sex of the baby, age of mother, father’s education, availability of CEmOC services, or if the mother used antenatal services four or more times were included in the analysis using logistic regression, and the results are presented in Table 4 as both unadjusted and adjusted odds ratios (both with 95% confidence intervals).

**Table 4:** Crude and adjusted risk factors for stillbirths.

Variables		Stillbirths		Crude OR	95% CI	Adjusted OR	95% CI	P value
		Yes	No					
CEmOC service available	Yes	224	23908	0.77	0.63–0.94	0.67	0.54–0.83	<0.001
	No	171	14009	1.00				
Sex of baby	Boy	255	19668	1.69	1.37–2.08	1.62	1.31–2.01	<0.001
	Girl	140	18249	1.00				
Age of mother in years				1.00	0.98–1.03	1.01	0.99–1.04	0.25
Father education: grades completed				1.00	0.97–1.03	1.02	0.98 1.06	0.278
Mother education: grades completed				0.99	0.94–1.02	0.99	0.95–2.04	0.747
No reported illness		73	32761	1.00		1.00		
Illness during pregnancy		21	2157	4.36	2.55–7–19	1.30	1.02–1.65	0.035
Illness pregnancy and delivery		148	1655	40.13	29.96–54.07	11.27	7.10–17.90	<0.001
Problems during delivery		153	1342	51.16	38.22–68.90	52.52	39.46–69.91	<0.001
More than 4 antenatal controls	Yes	97	12182	0.69	0.55 0.87	0.77	0.60–0.98	0.034
	No	298	25735	1.00				

During the birth registration, the health extension workers were asked if there had been illnesses during pregnancy or complications during the delivery. As seen from Table 4, the adjusted OR for stillbirth was 52.2 (95% CI 39.46–69.91) if the HEW noted complications during delivery. Mothers living in areas with access to EmOC institutions (OR 0.67) and if they used four or more antenatal visits (OR = 0.77) had lower risk of stillbirths. Boys had higher stillbirth rates than girls.

## Discussion

In our study, which aimed to operationalise interventions to reduce maternal and neonatal deaths, and stillbirths, we found a large reduction (46%) in stillbirth rates. The stillbirth rates at the start (14.5/1000 births) were similar to a recent estimate from Ethiopia of stillbirths (13.8 per 1000 births) [1]. Our interventions aimed at a broad population coverage of a “continuum of care” approach and focused on providing essential antenatal and obstetric services in communities through health extension workers, at antenatal and health facility services [25]. Thus, in two provinces with increased coverage of essential basic and comprehensive obstetric services we noted decreases stillbirth rates while referrals to institutions increased. We also noted a decrease in stillbirth rates at institutions, probably reflecting that mothers reached the institutions earlier and the quality of services at the institutions improved.

However, our results differ from earlier studies which showed that focusing on antenatal services, or skilled birth attendants alone did not reduce stillbirths [10]. Possible reasons for such as discrepancy could be that in our intervention study emphasis was on providing integrated services on “the continuum of care” addressing how patients can be cared for from their homes to using CEmOC services [26]. Isolated interventions only focusing on antenatal services or skilled birth attendants without addressing the referral chain and reducing possible delays in seeking care, may not give the expected results [9].

Furthermore, the large areas we did the interventions are similar in demographics, health services, road access, and economic structure with most rural communities in Ethiopia. Thus, our findings can be generalised to communities with similar access to health services and socio-economic characteristics as found in large parts of rural Ethiopia, may also apply to developing countries at similar stages of health service access and socio-economic development. Nevertheless, because of specific local differences in health service quality, utilisation, and cultural contexts, the rate of skilled birth attendance may vary.

Our study used a “continuum of care” approach and focused on providing essential antenatal and obstetric services in communities through health extension workers, at antenatal and health facility services [25]. The example from Dirashe, which had a well-functioning maternal waiting area [15], shows that stillbirth rates can be reduced if mothers at risk stay near to a hospital and can get essential services in time. Although our data show that focusing on CEmOC and antenatal services reduces stillbirths, the study also underlines that illnesses during pregnancy and complications during delivery still represent important risk factors for stillbirths. Although we did not assess how serious these illness episodes were, our results suggest that further studies are needed to describe the illness episodes that pregnant women in countries such as Ethiopia experience. Our study suggests that obstetric care need still needs to be strengthened and should include the continuum of care from home to health facility.

Although distance to the health institutions was a predictor for maternal deaths [13], we did not find any association between increased risk for stillbirths and availability of transport. However, the remotely lying Bonke area, with restricted access to CEmOC services, had higher stillbirth rates even if the mothers reached a hospital, probably reflecting delays in access to CEmOC institution. This may suggest that time may be a more critical factor to reduce stillbirths than for maternal deaths.

Our line of reasoning about why we consider the reduction in stillbirths to be a result of the interventions are based on applying some of the Austin Bradford Hill’s criteria for assessing possible causal associations as we outlined in our earlier paper [13, 27]. Thus, we found that the effect size was large (OR = 0.46). Also, the reduction in stillbirths occurred across several of the subgroups included in our study. The decline in stillbirth rates occurred after the start of the interventions, and thus showing temporality between interventions and outcome.

This study was done in rural communities, and most of the stillbirths occurred at home. Earlier studies that aimed to reduces neonatal deaths found that it reduced stillbirths, but not so much neonatal deaths [28]. This suggests there could have been a misclassification, and that stillbirths were over-reported while neonatal deaths were under-reported. As our recording was done by HEW with limited clinical skills, our registration does not provide enough information to discuss this potential misclassification. Also, in some communities, including the ethnic groups in our study area, talking about stillbirths and neonatal deaths are cultural taboos, which could lead to under-reporting of stillbirths [29]. We believe that this under-reporting would affect all areas and years. The gestational age was based on history taking of last menstruation period, and this may also have introduced a potential classification error.

The current data show that the stillbirth rates were low, especially at the end of our study period in 2013. This low rate could be partly explained by some limits in our study: the coverage of birth registrations was 72%. Our validation studies show that deliveries taking place at remote areas, had increased maternal mortality rates and used ANC less compared with those that lived nearer to community centres where the health post was found. The current study further shows that the stillbirth rate in the Bonke province, where the access to CEmOC services was low and based on transportation to another hospital, had high stillbirth rates among those referred to a hospital. By not measuring stillbirth rates in the remote areas, we may thus have underestimated the stillbirth rates. This high stillbirth rate among institutional deliveries is thought to be caused by delays in getting comprehensive obstetric care. However, this selection bias is believed to be larger at the start of our intervention programme and we expect that it was reduced as the population coverage of the interventions increased. So, even if we underestimated the stillbirth rates, the trend in stillbirth decline could thus be larger that we measured.

Our study suggests that stillbirths can be reduced if the health services focus on improved obstetric care. However, since many deliveries still take place at homes, identifying deliveries with complications and refer them on time to good institutions may further decrease stillbirths.
